# Non-canonical functions of BCL-2 family members in energy metabolism and necrotic cell death regulation

**DOI:** 10.1080/15384101.2025.2484868

**Published:** 2025-03-27

**Authors:** Mohamed El-Mesery, Franziska Rudolf, Yannick Heimann, Georg Häcker, Thomas Brunner

**Affiliations:** a Biochemical Pharmacology, Department Biology, University of Konstanz, Konstanz, Germany; b Collaborative Research Center TRR 353 “Death Decisions”; c Department of Biochemistry, Faculty of Pharmacy, Mansoura University, Mansoura, Egypt; d Institute of Medical Microbiology and Hygiene, Medical Center, Faculty of Medicine, University of Freiburg, Freiburg, Germany; e BIOSS Centre for Biological Signalling Studies, University of Freiburg, Freiburg, Germany

**Keywords:** BCL-2, apoptosis, mitochondria, electron transport chain, glycolysis, mitotoxicants

## Abstract

The large family of BCL-2 proteins plays a well-established role in the regulation of mitochondrial apoptosis pathway, and the crosstalk between death receptor signaling and mitochondrial apoptosis. Accumulating evidence suggests, however, that various BCL-2 family members are also involved in the regulation of apoptosis-unrelated necrotic forms of cell death, and even non-cell death processes. In this review, we discuss the emerging role of BCL-2 family members, and in particular BIM, in the regulation of mitochondrial dynamics, morphology and energy metabolism, and associated consequences for drug-inuced necrotic cell death.

## Introduction

The name of BCL-2 protein refers to the B cell lymphoma-2 gene, which encodes the first discovered protein of the BCL-2 family (BCL-2). BCL-2 was found to be overexpressed in human follicular lymphomas as a consequence of a t(14;18) chromosomal translocation bringing the *BCL-2* gene under the transcriptional control of the strong immunoglobulin heavy chain enhancer [1]. Subsequently, BCL-2 was recognized to be overexpressed not only in follicular lymphomas, but in a large number of different types of tumors [2,3]. Later on, close homologs of BCL-2 with conserved domain strcutures, referred to as BCL-2 homology (BH) domains, have been identified, leading to the establishment of the BCL-2 family [4,5]. BCL-2 proteins have important functions in different cellular processes. While their role in the regulation of apoptotic cell death is well established and represents one basis of modern cancer therapy, already early on additional activities of different family members in the regulation of cell cycle, mitochondrial metabolism, calcium homeostasis, glucose and lipid metabolism, and inflammation had been described [6]. Current research particularly emphasizes their roles in the control of mitochondrial morphology and functions. Their participation in the regulation of mitochondrial energy metabolism, generation of reactive oxygen species and “necrotic” cell death in the context of drug-induced liver injury will be discussed in this review.

## Canonical functions of BCL-2 family members: regulation of intrinsic apoptosis

The regulation of intrinsic apoptosis signaling pathways is considered the canonical function of BCL-2 family proteins, and their role is a critical decision-making step between cell survival and (apoptotic) cell death [4,7]. Based on the homologies in their protein structures and their distinct roles in apoptosis regulation, BCL-2 family members are classified into two main groups, anti-apoptotic members, including BCL-2, MCL-1, BCL-x_L_, BCL-w, and Bfl-1/A1, and pro-apoptotic members, which are further subdivided into executioner proteins, including BAK, BAX and possibly BOK, and so-called BH3-only proteins, including BIM, BID, BAD, BMF, NOXA, PUMA and HRK [4,5]. Key structural features to the BCL-2 family are their distinct protein domains, in particular the BH3 domain, enabling multiple interactions with other BCL-2 family members, ultimately regulating the balance between pro-survival and pro-apoptotic signals, and the final fate of the cell. The BH3 domains bind to the hydrophobic pockets of other BCL-2 members, enabling protein–protein interactions, and as a consequence neutralization or activation. The different BCL-2 family members are differentially expressed in different cell types and tissues, and differentially contribute to the execution of apoptotic cell death in these cells. An example is the important role of the BH3-only protein BIM (BCL2L11) in lymphocytes, as BIM deletion results in defective thymic selection, and development of lymphoproliferative and autoimmune diseases [8–10].

**Figure 1.**
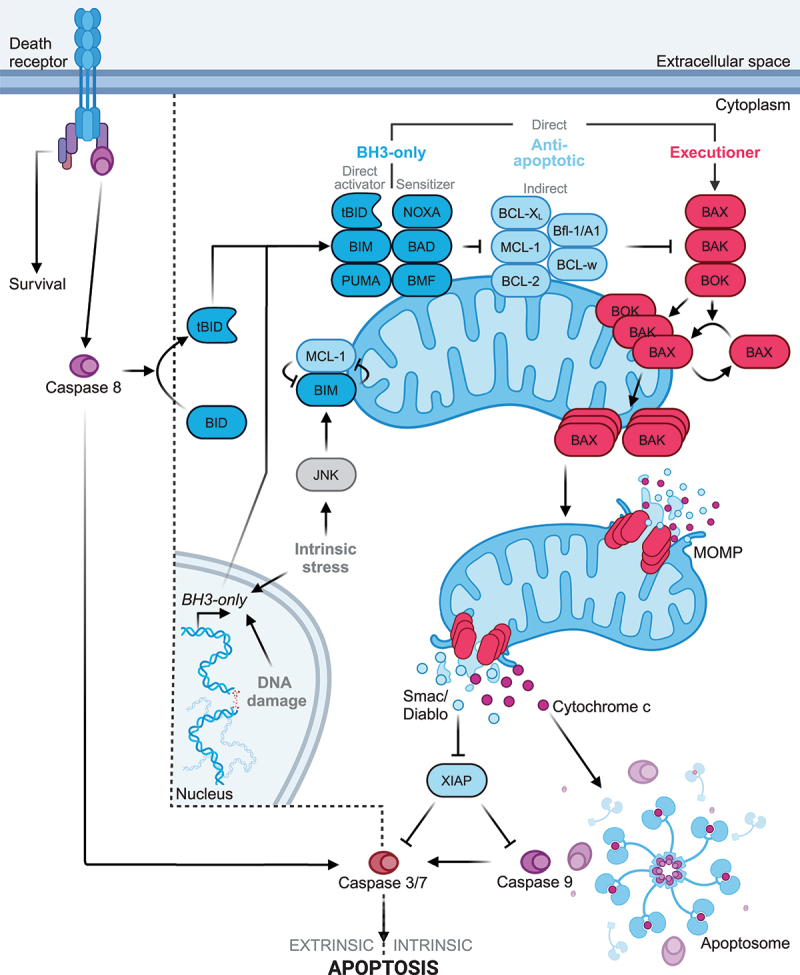
Canonical functions of the BCL-2 family. The canonical BCL-2 protein function, i.e. apoptosis, is triggered by extracellular and/or intracellular stimuli, which disturb the balance between pro- and anti-apoptotic BCL-2 proteins. Extracellular stimuli start with death receptor activation (such as TNF-R1, FAS and TRAIL-Rs), leading to DISC assembly and caspase 8 activation. Activated caspase 8 either directly activates the effector caspases 3 and 7, or triggers the intrinsic apoptosis pathway by inducing BID cleavage and the release of truncated BID (tBID). Intracellular stress stimuli affect the balance between BCL-2 proteins by transcriptional induction or posttranslational activation of BCL-2 proteins. There are two modes how the pore-forming members BAX and BAK become activated, the direct and indirect way. In the indirect way, anti-apoptotic proteins are neutralized by activated or overexpressed BH3-only proteins, which triggers BAX/BAK oligomerization and MOMP induction. The direct mode requires direct activation of BAX/BAK by certain BH3-only proteins to induce MOMP, which is antagonized by anti-apoptotic BCL-2 proteins. MOMP induction leads to the release of different mitochondrial proteins into the cytoplasm, such as cytochrome c and SMAC/DIABLO. Cytochrome c release induces apoptosome assembly, which triggers caspase 9 activation, subsequent activation of the effector caspases 3 and 7, and apoptosis induction.

It is generally accepted that various apoptosis-initiating signals either promote transcriptional upregulation of BH3-only proteins, or their activation via posttranslational modification, such as phosphorylation or proteolytic cleavage, although there are pro-apoptotic signals that do not lead to clearly observable BH3-only protein induction. Specific stress signals appear to target specific BH3-only proteins or groups of them. Their increased expression and/or activity enables them to bind via their BH3 domains to anti-apoptotic BCL-2 members and neutralize them, while a specific subset of BH3-only proteins (i.e. tBID, PUMA, BIM) is also able to directly activate the executioner members BAX and BAK, ultimately resulting in their oligomerization, the formation of protein pores and the permeabilization of the outer mitochondrial membrane (MOMP) (Figure 1). As a consequence, a number of mitochondrial proteins are released into the cytoplasm, initiating or supporting the activation of caspases, the key proteases in the execution of apoptotic cell death. Thus, cytochrome c binds to the adaptor protein APAF-1, which oligomerizes and recruits pro-caspase 9, resulting in formation of the apoptosome, a caspase activation platform. Autoproteolytic cleavage and activation of pro-caspase 9 initiates a caspase cascade and apoptosis execution (Figure 1) (reviewed in [11,12]).

Since their initial discovery, there has been a long-standing dispute how BH3-only proteins activate the pore-forming members BAX and BAK, and induce MOMP. Some favored the “indirect” model, in which increased levels or activities of BH3-only proteins result in binding to and neutralization of anti-apoptotic BCL-2 family members, thereby failing to neutralize BAX/BAK, consequently resulting in MOMP and apoptosis. Other scientists highlighted the capacity of certain BH3-only proteins to activate BAX/BAK in a “hit-and-run” manner in order for them to oligomerize and promote MOMP (“direct” model). The current understanding is that both activities can be relevant. It is clear from cells lacking many BCL-2-proteins that indirect activation occurs. The evidence is, however, strong that direct activation can also contribute [4] (Figure 1). The recognition of the importance of BCL-2 family interactions and the critical BH3 domain-mediated neutralization of anti-apoptotic BCL-2 proteins in the induction of mitochondrial apoptosis has led to the development of so-called “BH3 mimetics”, small molecular compounds, which act as inhibitors of anti-apoptotic BCL-2 proteins and represent already an established therapy in cancer patients (e.g. Venetoclax, ABT-199) (reviewed in [13]).

An important aspect to realize is that under steady-state conditions fully functional, viable cells already express a variety of different pro- and anti-apoptotic BCL-2 family member at different levels. Most notable is the activation-induced expression of BIM in neutrophils and T lymphocytes, which is not associated with cell death induction, but rather increased survival [14,15]. While their complex and dynamic interactions in the BCL-2 interactome may critically contribute to the neutralization of pore-forming members, the prevention of apoptosis and ultimately the survival of the cells, various BCL-2 proteins may have additional, non-canonical functions in the cells, which are unrelated to apoptosis regulation. Thus, similar to cytochrome *c*, whose “daytime” job inside the mitochondria is the generation of ATP as part of the respiratory chain (electron transport chain, ETC), and only when released into the cytoplasm it executes its “nighttime” job by promoting apoptosome assembly and caspase activation, also various BCL-2 family members may have “daytime” jobs in viable cells.

## Non-canonical functions of BCL-2 proteins

Defining non-canonical (i.e. apoptosis-unrelated) functions of BCL-2 proteins remains challenging. Numerous reports have suggested such functions, but the currently available data are oftentimes contradictory. Early on after the initial discovery of the *BCL-2* gene and its protein product [16–18], and its first association with programmed cell death regulation [19], mechanistical studies aimed to understand how BCL-2 inhibits programmed cell death. Early investigations also found nuclear functions of BCL-2 homologs [20], which can by now be regarded to as non-canonical functions. These studies revealed multiple interactions of BCL-2 proteins with nuclear proteins, either involved in DNA damage repair or cell cycle regulation. BCL-2 was reported to protect early mitotic cells, while decreased late mitotic BCL-2 expression sensitized cells to apoptosis [21]. Furthermore, BCL-2 was associated with enhanced nuclear and mitochondrial DNA repair after oxidative damage [22] and modulation of transmembrane trafficking, especially with regards to nuclear import of p53 [23]. Other BCL-2 proteins have also been reported to possess non-canonical functions in nuclear processes. MCL-1 is supposedly recruited to sites of DNA damage, and MCL-1-deficient cells show signs of delayed Chk1 and H2AX phosphorylation, and a suppressed DNA damage response [24], while overexpression of MCL-1 conveys S-phase arrest [25]. Furthermore, BCL-x_L_ was found to interact with Cdk1 and to inhibit its kinase activity [26,27]. On the other hand, the pro-apoptotic BCL-2 full-length protein BID has been shown to partially localize inside the nucleus and to be targeted by the DNA damage response kinase ATM after introducing DNA double strand breaks by ionizing radiaton, which was important to mediate S-phase arrest [28].

Apart from various nuclear functions of different BCL-2 proteins, proposed non-canonical functions of BCL-2 proteins range from regulating calcium homeostasis, autophagy, unfolded protein responses to cell migration. Many of these different non-canonical functions have been reviewed elsewhere (e.g. reviewed in [29–31]). In this review, we will thus primarily focus on non-canonical functions of BCL-2 family members in regulating mitochondrial morphology and energy metabolism.

## BCL-2 proteins and non-canonical functions in mitochondria

Most BCL-2 family proteins primarily localize to mitochondria. Thus, most BCL-2 family members (though not all) have domains that target them to the outer mitochondrial membrane, but which may also enable interactions with other membranes. Masking these hydrophobic domains by protein-protein interactions also enables the presence of BCL-2 proteins in the cytosol in the absence of membranes. For example, the transmembrane domain at the N-terminus of BAX is sequestered by its hydrophobic groove, and only activation by BH3-only proteins and/or other signals results in the unfolding of the transmembrane domain and insertion into the outer mitochondrial membrane [32]. Similarly, BCL-x_L_ dimers can redistribute to the cytosol, and BCL-x_L_ contributes to the retrotranslocation of BAX and BAK from the mitochondria to the cytosol [33,34]. Mitochondrial BCL-2 proteins are involved in mitochondrial homeostasis, including regulation of mitochondrial morphology, network formation by fission/fusion and energy metabolism. The BCL-2 family proteins mainly localize to the outer mitochondrial membrane, but also the inner mitochondrial membrane or the mitochondrial matrix. The topic of BCL-2 protein localization will be further discussed below.

**Figure 2.**
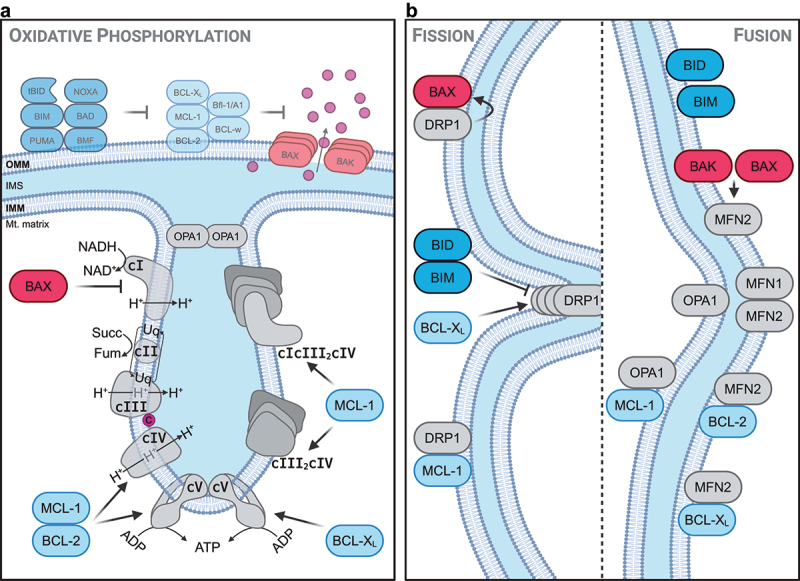
Non-canonical functions of BCL-2 homologs in mitochondrial metabolism and morphology. (a). In addition to their canonical functions, BCL-2 proteins excert non-canonical functions at the mitochondrial cristae by regulating mitochondrial respiration through interaction with complexes of the electron transport chain (ETC). Anti-apoptotic BCL-2 homologs positively regulate the ETC, via interaction with complex IV (cIV), complex V (cV) or by promoting the formation of supercomplexes (cIII_2_cIV, cIcIII_2_cIV). The pore-forming BCL-2 protein BAX is a negative regulator of the ETC by inhibiting complex I (cI). (b). BCL-2 proteins regulate mitochondrial morphology and function through fission and fusion. BID and BIM can inhibit DRP1 oligomerization-mediated fission, while BCL-x_L_ can enhance it. BAX and BAK interact with MFN2 and promote mitochondrial fusion. Yet to be confirmed is the exact molecular role of interactions of BCL-2 proteins with regulators of mitochondrial morphology, such as that of MCL-1 with DRP1 and OPA1, or BCL-2 and BCL-x_L_ with MFN2. The role of BID and BIM in mitochondrial fusion processes remains unknown.

The mitochondria are an important source of cellular energy production via the synthesis of ATP within the electron transport chain (ETC). In fact, many cell types, such as hepatocytes, primarily depend on oxidative phosphorylation to maintain their cellular energy levels [35,36]. The ETC is localized in the mitochondrial cristae (Figure 2(a)). Yet, BCL-2 proteins primarily localize to the outer mitochondrial membrane. It is thus somewhat surprising that interactions between BCL-2 proteins with different ETC proteins have been reported. BCL-2 has been shown to interact with complex IV and to regulate its efficiency. It was suggested that under steady-state conditions BCL-2 increases cytochrome c oxidase (complex IV) activity, while under stressed conditions it can inhibit complex IV in a S70 phosphorylation-dependent process, and thereby reduce ROS levels to attenuate cellular stress [37,38]. In detail, BCL-2 overexpression increased complex IV activity in CEM cells by improving the mitochondrial targeting of complex IV subunits via a direct interaction of BCL-2 with COX5A [39]. In neurons, BCL-x_L_ reportedly increases the efficiency of the ETC by closing a proton leak at the F_1_F_0_-ATPase, thereby increasing ATP production [40]. Tumor cells overexpressing BCL-2 or BCL-x_L_ have been found to be more resistant to metabolic stress due to a more efficient coupling of the proton motif force to ATP generation by the F_1_F_0_-ATPase [41]. Along the same lines, MCL-1 deletion reduced mitochondrial ATP production by disrupting the formation of supercomplexes in the ETC, which was found to be caused by a reduction in mitochondrial DNA content [42]. In addition to its effects on the ETC and mitochondrial ATP production, MCL-1 has also been reported to be involved in fatty acid metabolism via its interaction with either the acyl-CoA-synthetase ACSL1 [43] or the very-long-chain acyl-CoA dehydrogenase VLCAD [44]. Thus, it appears that especially the above mentioned anti-apoptotic BCL-2 proteins positively regulate the efficiency of the ETC, ATP production and thereby the cellular energy status, and allow for quick adjustments upon cellular stress to maintain mitochondrial homeostasis (Figure 2(a)).

On the other hand, the pro-apoptotic, pore-forming BCL-2 proteins BAX and BAK have been reported to exert cell type-specific effects on mitochondrial respiration. BAX/BAK double-deficient cells show increased or decreased mitochondrial respiration, depending on the cell type-specific modulation of the mitochondrial transcription elongation factor TEFM [45]. BAX has been shown to interact with and to inhibit the ND5-subunit of complex I, thereby inhibiting ROS production and associated tumor progression, which could be reverted by certain anti-apoptotic proteins [46–48] (Figure 2(a)).

## BCL-2 proteins and mitochondrial morphology

Mitochondrial energy metabolism is closely linked to mitochondrial morphology, and there is considerable crosstalk between the energetic phenotype (oxidative phosphorylation versus glycolysis) and the mitochondrial morphology of a cell. Given that BCL-2 proteins regulate mitochondrial energy metabolism, it is also expected that one of their non-canonical functions may be the regulation of mitochondrial morphology. Mitochondrial shape is largely regulated by mitochondrial fission and fusion. Indeed, various BCL-2 proteins have been implicated in these processes (Figure 2(b)). However, unlike their pro- or anti-apoptotic role, it is not possible to clearly group BCL-2 proteins into pro-fusion or pro-fission members, since currently available data greatly differ in this regard.

Almost all studies investigating the impact of BCL-2 proteins on mitochondrial morphology describe interactions with the mitochondrial fusion proteins mitofusin 1/2 (MFN1/2), the mitochondrial fission protein dynamin-related protein 1 (DRP1) and the mitochondrial fusion and cristae-organizing protein dynamin-like GTPase (OPA1) [49]. These interactions appear to be evolutionary conserved, since the *C. elegans* BCL-2 homologue CED-9 has been shown to induce mitochondrial fusion in *C. elegans* via the interaction with FZO-1 (MFN1/2 homolog) and EAT-3 (OPA1 homolog) [50], but is also able to induce mitochondrial reorganization in mammalian cells via its interaction with MFN2 [51]. Interestingly, at the same time CED-9 was not able to protect mammalian cells from various cell death stimuli [51], indicating that the effect of CED-9 on the mitochondrial fission/fusion process may not be dependent on interactions with other BCL-2 family members. The same study also showed interactions of mammalian BCL-2 and BCL-x_L_ with MFN2, which might be associated with mitochondrial aggregation observed in HeLa cells in response to BCL-x_L_ overexpression [52]. In a neuronal context, however, BCL-x_L_ positively regulated the MFN2-antagonist DRP1 to increase neuronal synapse formation [53]. Another study raised the notion that BCL-x_L_ can at the same time promote mitochondrial fission and fusion, but found an elongated mitochondrial phenotype in BCL-x_L_-overexpressing cells due to a BCL-x_L_-induced increase in mitochondrial biomass [54]. Along the same lines, MCL-1 has been reported to interact with DRP1 as well as OPA1, thereby having an impact on both fission and fusion events to regulate mitochondrial dynamics [55]. In addition, the interaction of MCL-1 with DRP1 has been shown to promote mitochondrial fragmentation in cardiomyocytes as a pro-survival reaction in response to different stressors [56]. Especially in the heart, various findings support an important role of non-canonical functions of MCL-1 in the regulation of mitochondrial morphology, fatty acid metabolism and autophagy. This may also contribute to the observed cardiotoxicity in a recently stopped clinical phase I trial with small molecule inhibitors of MCL-1 [57–59].

During apoptosis, mitochondria undergo extensive mitochondrial fission. It was thus somewhat unexpected that under steady-state conditions the pore-forming BCL-2 proteins BAX and BAK promote mitochondrial fusion via MFN2 activation [60]. BAX can, however, also interact with DRP1, and this interaction supposedly increases during apoptosis (Figure 2(b)). It has been even suggested that DRP1 might be an uncommon BH3-only-like activator of BAX [61]. Full-length BID has also been shown to play an apoptosis-unrelated role in the regulation of mitochondrial morphology, since BID-deficient cells show impaired formation of mitochondrial cristae structure [62]. This represents an excellent example of non-canonical, apoptosis-unrelated functions of a BCL-2 protein, as full-length BID, in the absence of caspase 8-mediated cleavage and apoptosis, appears to mediate this effect on mitochondrial morphology, while predominantly truncated BID (tBID, caspase-cleaved) neutralizes anti-apoptotic BCL-2 proteins and promotes BAX activation.

While the substantial number of publications makes clear that various BCL-2 members do indeed play a critical role in the regulation of mitochondrial dynamics and associated mitochondrial energy metabolism, reports are often contradictory. Given the already complex interaction patterns within the BCL-2 family (the BCL-2 interactome) [63], and considering a yet unknown new dimension of interactions with non-BCL-2 proteins, it is very likely that diverging outcomes on mitochondrial morphology and energy metabolism may be due to dynamic, context-dependent and cell type-specific formation of protein complexes, that could serve to maintain mitochondrial homeostasis during varying conditions.

## BIM, mitochondrial energy metabolism and necrotic cell death

Compared to the anti-apoptotic members of the BCL-2 family, the non-canonical functions of BH3-only proteins are rather little explored. Yet, recent data from our own lab provide strong evidence for a novel non-canonical, i.e. apoptosis-unrelated, function of the BH3-only protein BIM in the regulation of mitochondrial energy metabolism and morphology [64]. BIM is a BH3-only protein with up to 12 different isoforms generated by alternative splicing, although only the three major isoforms BIM_EL_ (extra-long), BIM_L_ (long) and BIM_s_ (short) are consistently detected on a protein level in cells [65,66]. Interestingly, while apoptosis-promoting stimuli sometimes induce BIM transcription [8,67], all isoforms are expressed at moderate to high levels even under steady-state conditions without promoting apoptosis, suggesting non-canonical functions of BIM in healthy viable cells [64]. Subcellular fractionation and colocalization experiments revealed that BIM isoforms and their anti-apoptotic binding partners are primarily localized at mitochondria, indicating that also BIM’s potential non-canonical functions under steady state conditions may be related to mitochondrial biochemical processes [65,68]. In healthy cells, the three isoforms of BIM are kept in an inactive status in protein complexes, but are released by stress and death signals. Two potentially different mechanisms have been described how BIM is maintained in an inactive (i.e. not apoptosis-promoting) form. An initial study identified dynein light chain 1 (DLC1) protein as a BIM interactor, and suggested that DLC1 interaction would sequester BIM to the cytoskeleton-associated motor complexes [69]. However, subsequent studies revealed that DLC1 forms high molecular weight complexes with BIM at the outer mitochondrial membrane [70]. Another mechanism, how the pro-apoptotic activity of BIM isoforms is hold in check, is via the interaction with anti-apoptotic BCL-2 homologs, such as MCL-1 and BCL-x_L_ [65,67]. In fact, MCL-1 constitutively interacts with BIM, and is also part of the high molecular weight protein complex with DLC1 [70]. Thus, the two BIM-inactivating mechanisms may be at least partially overlapping.

Since metabolism and cell death are connected, the cell’s ability to sense nutrients and metabolize different carbon substrates is not only important for anabolic and catabolic needs, but likely also contributes to the modulation of cell fate and function [71,72]. Interestingly, already under steady-state conditions, i.e. in the absence of apoptosis-promoting signals, BIM deficiency in hepatocytes promotes mitochondrial fragmentation/fission and increased cellular ATP production. Higher ATP levels are attributable to an increase in both, mitochondrial oxidative phosphorylation and glycolysis, and in general the cell’s metabolism is shifted toward a more glycolytic energy profile [64]. This interpretation is further supported by the observation that cells characterized by a glycolytic energy profile demonstrate increased fragmentation of the mitochondrial network, while cells predominantly relying on the more efficient mitochondrial oxidative phosphorylation exhibit elongated and fused mitochondria [73,74]. This shift in cellular energy metabolism from a predominant dependency on mitochondrial energy production in wild-type hepatocytes toward a more glycolytic energy profile in BIM-deficient cells has also been shown to be protective in drug-induced liver damage [64]. Paracetamol (acetaminophen, APAP) is a frequently used analgesic anti-pyretic drug. Although generally safe, when overdosed APAP leads to severe liver damage, which is characterized by a profound drop in cellular ATP levels to due mitochondrial damage and inhibition of the respiratory chain [75,76]. APAP-induced liver damage is characterized by classical oncotic necrosis, in the absence of caspase activation and apoptosis [76,77]. Most recently, we identified oxidative stress-mediated oxidation of cysteine residues in caspases as the underlying mechanism of caspase inactivation during APAP-induced liver damage [77]. Surprisingly, however, APAP treatment of mice or isolated hepatocytes results in the rapid and massive upregulation of the pro-apoptotic BCL-2 homologs BIM, PUMA and NOXA [64,78]. Even more astonishingly, although APAP-treated hepatocytes die by oncotic necrosis in the absence of any signs of apoptosis [77], BIM deficiency profoundly protects from APAP-induced liver damage [64,79]. These surprising findings thus imply that also BIM may have additional non-canonical functions in the regulation of drug-induced liver damage.

A potential role of BIM in the regulation of energy metabolism had been suggested before by *Wali et al*., who reported that BIM deficiency in mice affects body weight, fat deposition and energy metabolite abundance [80]. More efficient glucose uptake and reduced hepatic glycogen storage of BIM-deficient mice suggested a higher demand for glucose [64,80]. Analysis of the activity of mitochondrial respiratory chain complexes I-IV revealed a twofold increase in the complex IV activity in BIM-deficient liver tissue, while other complexes seemed not to be affected. This suggests a specific effect on, resp. interaction with the functionality of complex IV, rather than on the entire ETC machinery. Moreover, this effect is likely not mediated by an enhanced expression of the complexes of the respiratory chain or the assembly of complex I/II/IV supercomplexes in BIM-deficient hepatocytes [80].

BIM most likely regulates mitochondrial respiration and ATP production through interaction with other members of the BCL-2 family at the mitochondrial membrane, especially with anti-apoptotic members, as BCL-2, BCL-x_L_ and MCL-1 are already known to regulate mitochondrial membrane potential [81,82], fission and fusion [42,83,84], oxidative phosphorylation [40,42,85,86] as well as mitophagy [87]. Briefly, it was shown that BCL-2 is able to bind and hyperactivate complex IV, whereas BCL-x_L_ and MCL-1 have already been discussed above (Figure 2).

Currently, it is still unknown whether BIM is able to directly bind complex IV or if its deletion increases the availability of anti-apoptotic BCL-2 members in the mitochondrial matrix [80]. But high-affinity interactions of BIM with pro-survival members and their sequestration might impact their non-canonical functions on the mitochondria [88]. Consequently, their non-canonical activities might be unmasked in the absence of BIM. Interestingly, BIM-deficient cell lines, hepatocytes and mouse liver tissue were shown to express higher levels of Hexokinase 1 (HK1), correlating with increased glycolysis [64]. This is supported by the finding that HK1 and 2 are associated with the mitochondria, enabling accelerated glycolytic ATP production due to an enhanced availability of ATP for the first step of glycolysis converting glucose to glucose-6 phosphate, which is ATP-dependent [89,90]. Furthermore, it has been described that BIM directly interacts with Hexokinases at the mitochondria, where they mutually modulate their respective activities [91–93]. Recently, a direct interaction between BIM and Hexokinase 3 (HK3) could be demonstrated in acute myeloid leukemia (AML) cells. HK3 can be distinguished from its isoforms HK1 and HK2 by the absence of a 21 amino acid sequence in the N-terminal region, allowing HK1 and HK2 to localize to the outer mitochondrial membrane, suggesting involvement of distinct HK isoforms in different processes [91]. Furthermore, it was proposed that the interaction between BIM and HK3 might lead to a phosphorylation-dependent degradation of BIM. Loss of HK3 resulted also in increased ROS levels, and since recent data from our lab demonstrated that loss of BIM reduces ROS production, it can be speculated that HK3 might also interfere with BIM-regulated ROS production [64,91]. However, given that HK3 is predominantly cytoplasmic, whereas BIM, HK1 and HK2 are localized to the mitochondria, the conditions of potential physical interactions between BIM and Hexokinase isoforms, and associated consequences for the cellular energy metabolism, respectively ROS production, need to be investigated in more detail. As discussed above, increased BIM expression has been reported in activated neutrophilic granulocytes or T lymphocytes [14], in the absence of apoptosis induction. Neutrophil activation is known to be accompanied by increased ROS production [94], which leads to the suggestion that activation-induced BIM expression might contribute to the elevated ROS levels, given that BIM deficiency lowers ROS levels [64]. Alternatively, lower mitochondrial ROS levels in BIM-deficient hepatocytes might be also mediated by non-sequestered pro-survival BCL-2 family members, since BIM was shown to inhibit the interaction between BCL-2 and glutathione (GSH) at the mitochondria, an important antioxidant and scavenger of mitochondrial ROS [95].

## BIM and mitochondrial dynamics

As outlined above, mitochondrial dynamics, including mitochondrial fusion and fission, are core processes of mitochondrial quality control and are closely linked to bioenergetic adaptations to metabolic demands [71]. Mitochondrial fusion is regulated, among others, by MFN1/2 and OPA1, whereas proteins like DRP1 and mitochondrial fission 1 protein (FIS1) are involved in the fragmentation (fission) of the mitochondrial network (Figure 2(b)) [96,97]. Although BIM does not appear to directly intseract with Mitofusins or DRP1 [64], DRP1 and MFN1/2 were found to co-localize with several other members of the BCL-2 family at the outer mitochondrial membrane, indicating a mutual regulation of fission/fusion processes based on their physical interaction [83]. This suggests that BIM deficiency-related mitochondrial fragmentation might be at least partially due to interactions of pro-survival BCL-2 proteins with mitochondrial fission/fusion-regulating proteins, as downregulation of the pro-survival proteins BCL-2, BCL-x_L_ and MCL-1 causes mitochondrial fusion and increases mitochondrial volume [83,84,98]. Interestingly, the pro-survival isoform of MCL-1, which is able to bind all BIM isoforms and which localizes via its transmembrane domain to the mitochondria, serves as a molecular anchor for mitochondrial DRP1. A reduction of MCL-1 at the outer mitochondrial membrane may limit the activity of DRP1 resulting in mitochondrial hyperfusion. Conversely, it can be assumed that an increase of free MCL-1 in BIM-deficient cells might result in an enhanced mitochondrial fragmentation in a DRP1-mediated manner [99,100].

Despite lacking evidence of direct association of BIM with MFN1/2 or DRP1, BIM and also BID were shown to promote the disassembly of OPA1 oligomers in a BH3 domain-dependent manner. Interestingly, no such activities could be shown for BAD, NOXA, BCL-2 or BCL-x_L_ [101], suggesting that specific BCL-2 homologs mediate specific non-canonical activities. OPA1 overexpression enhances the stabilization of the oligomeric ATP synthase, whereas the deletion of OPA1 decreases the abundance of ATP synthase. Instead, an increase in free F1 subunits, and a reduction in ATP synthase activity as well as total protein levels can be observed. Thus, OPA1 levels correlate with the stabilization and oligomerization of the ATP synthase [102,103]. Therefore, it appears reasonable to assume that BIM deficiency stabilizes OPA1 oligomers, and thereby enhances mitochondrial-driven ATP production. Furthermore, the shape of the cristae is a central morphological parameter affecting mitochondrial respiration, which is strongly affected by OPA1. OPA1 oligomers restrict cristae widening and thereby stabilize the respiratory supercomplexes to overall increase respiratory efficiency [103] (Figure 2).

Interestingly, it had been discussed that the BH3-motif of BIM may not have evolved by an evolutionary relationship with the ancient BCL-2 family, but rather by convergent evolution, suggesting that BIM’s non-apoptotic function could also be different from other BH3-only proteins [104]. Autophagic removal of cellular organelles appears to be more than an essential survival mechanism during nutrient deprivation by causing the degradation of cytosolic components. Rather, it is regarded as a central element in cellular homeostasis by removing superfluous or dangerous entities [105,106], including damaged mitochondria via so-called mitophagy. Thus, it should be noted that BIM appears to be involved in autophagy inhibition by sequestering Beclin to the dynein light chains. This is mediated through direct protein-protein interaction of BIM and autophagy-regulating protein Beclin, and seems not to require BCL-x_L_-Beclin interaction. In addition, BIM and BCL-x_L_ appear to interact with Beclin via different regions. In BIM-deficient cells, Beclin is presumably not bound to inhibitory complexes, which may permit mitophagy induction. Interestingly, BIM overexpression efficiently suppressed autophagy, whereas autophagy was enhanced in BIM-deficient cells [67,104]. In contrast to BIM, other BH3-only proteins appear to rather have a pro-autophagic role by releasing Beclin from the inhibitory anti-apoptotic complex [6]. More evidence for an autophagy-suppressing role of BIM is provided by our recent finding that BIM deficiency across different cell types and tissues results in a massive upregulation of the mitophagy-regulating enzyme Parkin and an induction of mitochondrial biogenesis gene expression [64]. Together with the enhanced fragmentation of the mitochondrial network, it can be suggested that BIM-deficient hepatocytes have a higher mitochondrial turnover via increased mitophagy and mitochondrial biogenesis. Very likely these processes contribute also to a reduced sensitivity of BIM-deficient hepatocytes to various mitochondria-damaging agents, such as APAP and complex I inhibitor rotenone [64].

## BIM phosphorylation and cellular energy status

While BIM proficiency versus deficiency clearly represent two most extreme stages of the BIM protein expression status in a cell, it can be speculated whether intracellular signaling processes, which affect BIM protein levels and/or activities may also contribute to BIM’s role in the regulation of mitochondrial fission/fusion and energy metabolism. Especially post-translational modification of BIM via phosphorylation through ERK1/2 and JNK are interesting in this regard. ERK1/2-mediated phosphorylation of BIM_EL_ at S69 (human) is known to target it to proteasomal degradation, ultimately reducing BIM protein levels [107]. Interestingly, the MAP kinase pathway and ERK1/2 are particularly activated upon growth factor receptor activation, such as the EGF or insulin receptor [108,109]. While it makes sense that cells activated by mitogenic stimuli may be protected from undergoing apoptotic cell death by reducing the levels of BIM, an ERK1/2-mediated reduction in BIM levels could also shift the cellular energy metabolism from oxidative phosphorylation toward glycolysis and the synthesis of building blocks for the further growth of the cell. Along these lines it is also interesting to note that BIM_EL_ becomes phosphorylated during onset of mitosis by the Aurora A kinase, resulting in the targeted degradation of BIM_EL_ [110]. While this reduction in BIM protein levels may ensure survival of the cell during mitosis, it may also bring the cell in an energetically more favorable situation to duplicate cellular components and divide fragmented mitochondria into the resulting daughter cells. In contrast, JNK is activated by various stress signals, which rather prime the cell toward apoptotic cell death. JNK phosphorylates BIM_EL_ at T116, a site which is known to interact with DLC1 [111]. Supposedly, phosphorylation of this site results in the release of BIM from the high molecular weight complex with DLC1, and the activation of apoptotic processes at the outer mitochondrial membrane. Interactions of BCL-2 family members with the mitochondrial fission/fusion machinery, the respiratory chain and associated ROS production may further tune BAX/BAK-mediated permeabilization of the outer mitochondrial membrane, apoptosome formation and caspase activation. Increased ATP production via the ETC would further support the apoptosome formation as a necessary co-factor. However, in the most extreme situation excessive mitochondrial ROS production may also result in the inactivation of the apoptosome or caspases by cysteine oxidation, as observed during APAP-induced hepatocyte necrosis [77]. This in turn could shift an immunologically silent apoptotic cell death into a necrotic type of cell death with the associated release of DAMPs (danger-associated molecular patterns) and the activation of immune cells.

## Not only BIM?

The literature on non-canonical functions of BCL-2 proteins discussed above suggests that many of the various pro-survival and pro-death BCL-2 family members directly or indirectly interact with other mitochondrial proteins, and thereby affect mitochondrial morphology and function. However, a clear grouping into specific functions remains difficult, especially for BH3-only proteins. In this regard, it is interesting to note that APAP-initiated stress signals in hepatocytes result in the simultaneous upregulation of BIM, PUMA and NOXA, and the activation of BAX [64,79,112]. As outlined above, deletion of BIM results in a profound protection of mice from APAP-induced liver damage [64]. Similarly, also deletion or inhibition of PUMA protects mice from APAP-induced liver damage [78]. In marked contrast, however, we have seen that deletion of NOXA rather accelerated the APAP-induced damage in the liver [64]. Similarly, the deletion of BAX resulted in reduced liver damage at early time points after APAP administration, however, no benefit over control mice was seen at later time points [113]. It is possible that activation of the other pore-forming member BAK may be able to compensate for the lack of BAX. Yet, the role of BIM and PUMA in APAP-induced liver damage may not solely be the activation of BAX and BAK, and induction of the outer mitochondrial membrane permeabilization, as during intrinsic apoptosis, but rather to regulate mitochondrial respiratory processes.

A particularly interesting case is the role of BID in APAP-induced liver damage. As discussed above, the excessive ROS generation during APAP-mediated mitochondrial damage results in the inactivation of caspases and in necrotic cell death [77]. Thus, processing of full-length BID to truncated tBID by caspase 8 does not occur during APAP-induced liver damage. Nonetheless, siRNA-mediated downregulation of BID resulted in a significant protection from APAP-induced liver damage [114]. This is surprising as full-length BID is mostly, though not exclusively, cytoplasmic and only tBID is known to interact with other BCL-2 family members to promote apoptosis. Thus, this finding clearly supports the notion that also full-length BID may have a “daytime” job in the regulation of mitochondrial energy metabolism and/or oxidative processes. However, this non-canonical role of BID in mitochondrial respiration would require mitochondrial localization of full-length BID. It is thus interesting to note that loss of BID results in apoptosis-independent irregular mitochondrial cristae formation in cardiomyocytes, associated with decreased mitochondrial respiration [62]. Furthermore, it has been found that the defect of BID-deficient cardiomyocytes could be rescued even by BID with a mutated BH3 domain, incapable of promoting apoptosis. Last, mitochondrial matrix localization of full-length BID was found to be independent of caspase 8-mediated processing, but rather to involve a lipophilic stretch in helix 6. Similarly, BID was recently also found to be involved in ferroptotic cell death induced by erastin, and to promote mitochondrial damage and fragmentation, associated with reduced ATP production [115]. Thus, very specific BH3-only proteins may have distinct roles in the regulation of mitochondrial fission/fusion processes and energy metabolism. Which role the respective BH3 domains play also in these processes remains to be investigated.

## Translation aspects of non-canonical functions of BCL-2 family members

**Figure 3.**
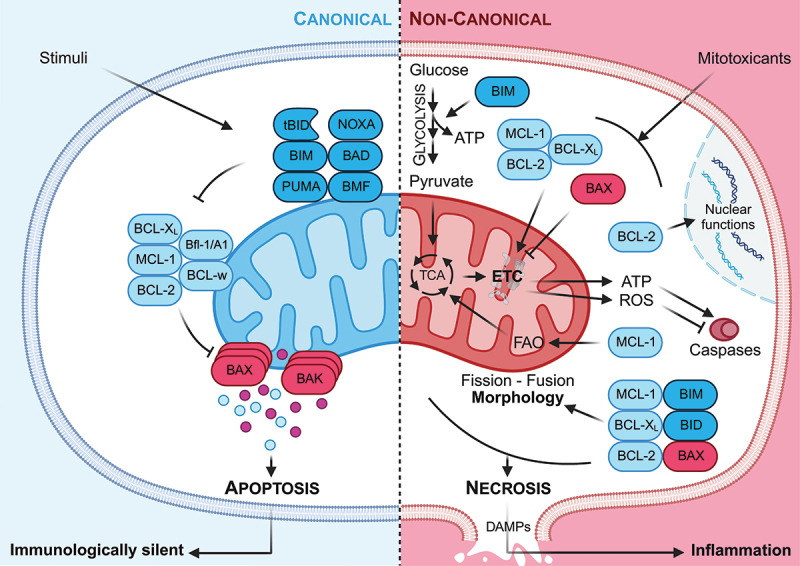
Summary of canonical and non-canonical functions of BCL-2 homologs. Cellular functions of BCL-2 proteins are divided into canonical and non-canonical functions. Canonical functions are related to MOMP induction and subsequent execution of apoptotic cell death, which is considered immunologically silent. Non-canonical functions include the role of BCL-2 proteins in energy metabolism, regulation of mitochondrial fission/fusion and ROS production. Mitotoxicants damage the mitochondria, inhibit the ETC, promote ROS production and induce expression of BH3-only proteins. BCL-2 family members regulate mitochondrial energy metabolism and fission/fusion. Since the apoptosome activation requires ATP, and ROS inhibits caspase activation, BCL-2 family members also regulate necrotic cell death and associated inflammation.

A classical distinction between apoptotic and necrotic cell death is the requirement of ATP for apoptosis and the permeabilization of the plasma membrane during necrosis. The assembly of the apoptosome and associated apoptosis induction requires sufficient ATP levels, and thus cellular respiration. In contrast, inhibition of mitochondrial respiration, for example after intoxication with sodium azide, complex I inhibitor rotenone, but also APAP, results in a massive drop in cellular ATP levels. As a consequence, the cellular salt and water balance is impaired, causing osmotic stress, cellular swelling, plasma membrane rupture and necrotic cell death. As during apoptosis plasma membranes of dying cells remain intact, the release of DAMPs is prevented and immune cells are not activated (Figure 3). In marked contrast, necrotic cells release various DAMPs, which results in inflammation. While plasma membrane rupture could promote inflammation and development of autoimmune diseases, and is likely to be deleterious in homeostatic cell death, e.g. during tissue renewal or thymic selection, a more inflammatory cell death during cancer therapy would be beneficial as it could enhance the therapeutic success due to induction of an anti-tumor immune response.

Overcoming cancer resistance to chemotherapy remains one of the greatest challenges in cancer therapy. Accumulating evidence demonstrates that downregulation of pro-apoptotic proteins or upregulation of anti-apoptotic proteins of the BCL-2 family contributes to the resistance against currently available anti-cancer drugs [2,116]. Therefore, inhibition or downregulation of the anti-apoptotic BCL-2 proteins is an attractive target for an effective cancer therapy. BH3 mimetics represent such a class of the anti-cancer drugs, which essentially mimic the action of BH3-only proteins and antagonize the inhibitory effects of anti-apoptotic Bcl-2 proteins [117]. Venetoclax (ABT-199) is an example of a BH3 mimetics and FDA-approved drug for the treatment of chronic lymphocytic leukemia. It is used either alone or in combination with other drugs, such as Obinutuzumab and Rituximab [7,118]. Unfortunately, patients often develop resistance against Venetoclax, which decreases its efficacy, and therefore further research is required to understand resistance mechanisms and to develop novel strategies to overcome cancer resistance and enhance treatment efficacy [119].

Indeed, apoptosis is not the only mode of cell death induced by BH3 mimetics in cancer cells. Recently, it was shown that BH3 mimetics induce caspase-independent cell death (CICD) in B cell lymphoma, that was associated with activation and recruitment of cytotoxic cells and increased release of inflammatory cytokines [120], suggesting increased release of DAMPs and associated induction of inflammation. Further investigation is still required to characterize the mode of cell death in different cancer settings and to understand the detailed mechanism of action. It is tempting to speculate, however, that the above discussed role of BCL-2 family members in the regulation of mitochondrial morphology and energy metabolism may at least in part contribute to this necrotic, inflammatory cell death. Thus, the BCL-2 family represents an attractive target for alternative therapeutic options in cancer therapy with the goal to enhance anti-tumor immune responses.

On the other hand, necrotic cell death and associated inflammatory responses are also known to promote progressive tissue damage in various diseases, including drug-induced hepatitis and arterial infarction. Thus, an immunologically silent cell death by apoptosis would be preferable over the uncontrolled spreading of necrosis. BCL-2 family members and non-canonical processes regulated by them could also represent interesting targets in the prevention and treatment of tissue damage by necrosis. Along these lines, it was interesting to note that the protective effect of BIM deficiency on APAP-induced liver damage could be simulated by shifting hepatocytes to glycolysis by high glucose administration, thereby maintaining sufficiently high ATP levels and permitting cellular recovery by induction of mitophagy or execution of apoptosis [64]. Thus, the increasing understanding of the role of various BCL-2 family members in regulating non-canonical functions in mitochondrial processes may allow to develop drugs, which could either prevent mitochondria-dependent necrotic cell death and thereby support tissue integrity, or on the contrary boost CICD to render cancer therapy more effective (Figure 3). A detailed future analysis of these processes will reveal both, risks and chances of BCL-2 family-based therapies in tissue homeostasis and cancer therapy.

In summary, as this review highlights various BCL-2 family members not only critically regulate mitochondrial apoptotic processes and thus cell death and survival (Figure 1) but via interactions and complex formation with other mitochondrial proteins also various homeostatic processes related to mitochondrial fission/fusion, morphology and energy metabolism (Figure 2). Excitingly, via the regulation of these non-canonical processes in the mitochondria the cell death-regulating activities of BCL-2 proteins appears not to be restricted to apoptosis but to extend also to necrotic forms of cell death (Figure 3).
